# Reviews and Book Notices

**Published:** 1879-10

**Authors:** 


### Reviews and Book Notices.

---

Article XIV.—Philosophical Problems in Medicine.—Presidential Address Before the American Medical Association, at its Thirtieth Annual Meeting, Atlanta, Ga., May 6, 1879. By Theophilus Parvin, m.d.

We have read this essay with great pleasure. The opening sentence of the address strikes its key-note: "Science, Literature, Philosophy and Theology are the great subjects of human study."

The aim of the author is the enforcement of the proposition that these four great provinces of investigation have almost equal claims upon the attention of the genuine scholar, and that no one can afford to plead devotion to any one or two of these fruits of the tree of knowledge as an excuse for neglect of the rest. It is very true that in all this there is nothing new; but every age and every generation of men needs the same counsel which was found needful for the preceding. Especially at the present time does such iteration seem necessary, for the great progress which has been recently made in certain branches of natural science has filled certain minds of the lower grade with an infatuated notion that physical science is pretty nearly all that one should know. The world is therefore filled with cries against waste of time in the study of literature, philosophy and theology. This, however, for the most part proceeds from those superficial students who merely skim the surface of science, compelling themselves to abide content with the observation of phenomena alone, to the utter suppression of all curiosity concerning their causes, which form the entire subject matter of philosophy and much of theology.

Dr. Parvin has done good service by showing in his own person an excellent example of the manner in which every genuine student opens his eyes to the facts of the universe. A great physician—a specialist in science—yet well informed in relation to all the other departments of human thought. In this way only is it possible for the highest perfection to be attained in this world. For want of this broad knowledge we everywhere see men stumbling along among the facts which form the basis of science, yet unable to see their connection, and missing entirely their highest signification. The knowledge of such men is full of lacunæ—vast spaces of darkness and silence—which they profess to hope will at some future time be illuminated by the progress of natural science, but which no amount of such progress alone can ever fill. It is the dry light of philosophy which is needed by these one-eyed giants, to reveal the golden statues in the darkened temple where they grope. Most fortunately, this light shines for all who desire its radiance; and we may hope that the time is at hand when it will be as fashionable to call for the "Man with the Lamp," as it now is to rest content with the indolent abnegations of a supercilious agnosticism. H. M. L.

ARTICLE XV. — SPERMATORRHŒA; ITS CAUSES, SYMPTOMS, RESULTS AND TREATMENT. By Roberts Bartholow, A.M., M.D., Professor of the Theory and Practice of Medicine and of Clinical Medicine in the Medical College of Ohio, etc., etc. Fourth edition, revised. New York: William Wood & Co., 1879.

Dr. Bartholow's book—originally a clinical lecture—has in this fourth edition, grown to a handsome monograph of 128 pages. Although the author is "more than ever confirmed in the view" that spermatorrhœa is a "*neurosis*," his methods of treatment are wonderfully like those employed by believers in the uniform existence of local causes. I. N. D.

